# Prediction of ship bulkhead deflection under internal explosion

**DOI:** 10.1038/s41598-026-43574-w

**Published:** 2026-03-13

**Authors:** Qing-hua Chen, Yan-guang Tao, Zhen-gang Liang

**Affiliations:** School of Equipment Engineering, Shenyang Ligong University, Shenyang, , 110159 Liaoning China

**Keywords:** Cabin internal explosion, Load simplification, Plastic deformation, Energy conservation, Engineering, Mathematics and computing, Physics

## Abstract

Internal explosions within ship compartments pose a severe threat to structural integrity. To investigate the plastic dynamic response of ship bulkheads under such loading, this study presents a comprehensive approach integrating experimental testing, numerical simulation, and theoretical derivation. First, a numerical model for shock wave propagation in a confined cabin was established to characterize the complex load distribution. Based on the simulation results, fitting formulas for reflected shock wave overpressure in different bulkhead regions were derived. Subsequently, a simplified theoretical model for predicting plastic deformation was developed based on the principle of energy conservation and the momentum theorem. By utilizing impulse equivalence and constructing a deflection shape function, the plastic dissipation energy—including the bending of plastic hinge lines and in-plane membrane stretching—was analytically solved. To validate the proposed theoretical model, cabin explosion tests using 80 g TNT charges were conducted. The results indicate that the theoretical predictions align well with the experimental measurements, with a relative error of approximately 13.8%. This demonstrates that the proposed method provides a rapid and effective tool for predicting the plastic deformation of bulkheads under internal blast loading, offering a practical alternative to time-consuming numerical simulations.

## Introduction

The penetration of anti-ship missiles through a ship’s side protection structures,followed by their detonation within internal compartments, can induce severe damage to the compartment and even the overall ship structure^1^. Within this internal explosion environment, the structure is subjected to two primary pressure loads: the shock wave and the quasi-static pressure^2^. Analyzing the dynamic response of structures to these internal loads is therefore a critical issue in the blast-resistant design of naval ships. Compartment walls encompass a variety of configurations, primarily constructed from fundamental unit components such as plain metal plates, stiffened plates, and laminated plates. Consequently, investigating the structural damage effects under internal explosions essentially equates to studying the response characteristics of these basic units to the ensuing blast loads^3^.

Extensive attention has been devoted by researchers worldwide to the characteristics of internal explosion pressure loading and the destructive behavior of ship bulkheads. Syrunin^4^ conducted a series of experimental studies in an explosion vessel, arranging sensors along the vessel wall to explore the shock wave propagation process, while Kong et al.^5^ systematically summarized the combustion enhancement effects and saturation impulse phenomena in confined internal explosions, providing a new perspective for load modeling. Xu et al.^6^ adopted improved numerical methods and simplified analytical models to systematically predict the blast loads in partially confined chambers, with a focus on the distribution of shock wave pressure and the evolution of quasi-static pressure. Jiang et al.^7^ carried out experimental and numerical simulations on the dynamic response of box-shaped structures subjected to internal explosions. They compared the peak pressures and impulses at different locations, revealing the concentration effect and non-similarity characteristics of internal blast loads in corner regions of the structure. Based on confined and partially confined explosion experiments, Rigby et al.^8^ proposed a theoretical model for predicting quasi-static pressure, which was applied to the blast response simulation of flexible structures, significantly improving the engineering applicability of load calculation. Wang et al.^9^ established a simplified model of internal blast loads for structural response analysis in fully confined spherical shell structures, and its predictions showed good agreement with empirical methods in design manuals. To further refine the spatial distribution description of the blast load, Chen et al.^10^ categorized the spatial distribution of internal blast loading into the non-corner central region, the dihedral corner area, and the trihedral corner area, and established a simplified computational model for the load on the target wall surface under cabin-internal explosion. Regarding research on structural dynamic response, Jones^11^ conducted a theoretical analysis of the dynamic response of simply supported circular plates under uniform shock loading, establishing a theoretical model based on energy conservation. Baker^12,13^ focused on fully clamped square target plates and derived the deflection calculation formula at the center of the square plate. Schleyer^14^ investigated the deformation of square plates under uniformly distributed triangular impulse loading by varying edge constraints, developing a dynamic model for plate deflection calculation. Rakvag et al.^15^ explored the dynamic response of target plates with pre-formed holes under shock wave loading, comparing the deformation of target plates with different hole shapes through experiments. Lee^16^ used numerical simulation software to analyze the deformation and deflection of fully clamped metal plates under varying local load intensities, determining the occurrence of tearing failure based on the ultimate strain of the target plate. Geretto et al.^17^ studied the failure modes of target plates under explosion loading in box-type structures, summarizing the effects of explosive charge mass on target plate plastic deformation under various confinement conditions by adjusting the degree of freedom constraints of the internal explosion environment.To further refine the spatial distribution description of the blast load. Zheng et al.^18^ comprehensively employed experimental and numerical approaches to systematically investigate the dynamic response of steel plates under confined blast loading. Using the energy method, they analyzed the plastic deformation mechanism of plates and established corresponding predictive equations for deformation. Zheng et al.^19^ studied the plastic deformation of metal reinforced plates under explosive loading, establishing a target plate deformation deflection calculation model based on the energy method and elastoplasticity theory, which was verified by experiments. Liu et al.^20^ established a deflection calculation model for reinforced plates based on the law of energy conservation and elastoplastic mechanics, conducting experimental validation. Liu ^21^ explored the anti-detonation mechanism of large-size laminated thin plates under proximity explosions of combatants, analyzing their deformation process, strain distribution, and deformation energy absorption, and demonstrated that laminated plates exhibit significantly improved deformation energy absorption compared to single-layer plates. Zhu et al.^22^ carried out extensive research on the saturation response of metal plates under explosive loading, investigating the effects of target plate structural dimensions, boundary constraints, material strain-rate sensitivity, and load impulse waveform on the saturated impulse law.

Despite these advances, existing simplified models often neglect the corner convergence effect of shock waves and the coupling of quasi-static pressure in confined cabins. To address these limitations, this study proposes a comprehensive prediction method. The main contributions are as follows: (1) Based on the spatial non-uniformity of blast loads, a zonal calculation model is established to quantify the shock wave overpressure in different bulkhead regions (central, near-wall, and corner); (2) A simplified theoretical model for plastic deformation is derived using the energy conservation principle, explicitly incorporating the strain-rate effect and the contribution of quasi-static pressure; (3) The proposed method is validated not only by our own experiments with varying structural stiffness but also by external independent datasets, proving its robustness for engineering rapid assessment.

## Internal explosive load modeling

### Numerical modeling and validation

**Fig. 1:**
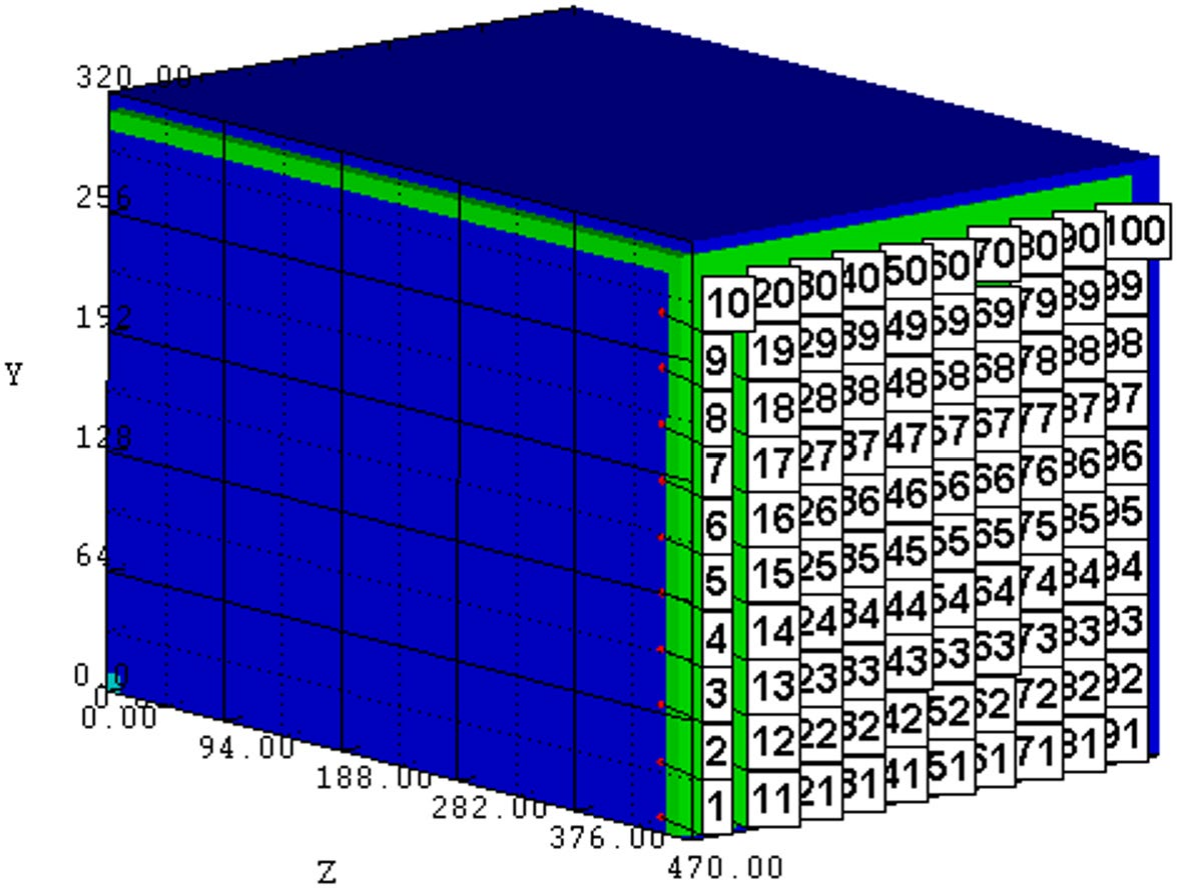
Numerical model.

To investigate the shock wave propagation characteristics in the cabin, a one-eighth scaled numerical model of the cabin is established (Fig. 1). The air domain is modeled using Eulerian elements, and the cabin structure is constructed with Lagrangian elements. The internal dimensions of the cabin are 900 mm × 600 mm × 600 mm, the bulkhead material is Q235 steel with a thickness of 15 mm. The Euler–Lagrange fluid–structure interaction algorithm is adopted between the air and the cabin wall. The outer boundary of the air domain is set as an outflow boundary, and after grid size optimization, the grid size for all materials in the model is determined as 5 mm.

TNT is selected as the explosive source, arranged at the geometric center of the cabin. Given that the explosive size is much smaller than the cabin dimensions, TNT is directly filled in the air domain to calculate the internal explosion shock wave load. The Jones-Wilkins-Lee (JWL) equation of state is used to describe the TNT explosive in the model:

1 $$P = A\left( {1 - \frac{w}{{R_{1} V}}} \right)e^{{ - R_{1} V}} + B\left( {1 - \frac{w}{{R_{2} V}}} \right)e^{{ - R_{2} V}} + \frac{wE}{V}$$

In Eq. (1): *P* is the pressure; *V* is the relative volume of explosive products; *E* is the initial energy density; *A*, *B*, *R*_*1*_ , *R*_*2*_ , *w* are explosive characteristic parameters (Table 1).

**Table 1:** Parameters of explosives.

Parameters	*A/*GPa	*B/*GPa	*R*_1_	*R*_2_	*w*	D/Km s^−1^	CJ/GPa
Numerical value	371.2	3.2	4.2	0.95	0.3	6930	21

The air is modeled as an ideal gas to ensure the general applicability of the calculation results and processes. The pressure per unit mass* P* is given by:

2 $$P = \left( {\gamma - 1} \right)\rho e$$

In Eq. (2): air density $\rho =1.225kg/{m}^{3}$; internal energy per unit mass of air $e=2.068\times 1{0}^{5}\hspace{0.17em}J/kg$; adiabatic index $\gamma =1.4$.

Q235 steel is selected as the structural material for the cabin, with its intrinsic parameters listed in Table 2.

**Table 2:** Structural material parameters.

Parameters	*ρ/g *cm^−3^	*A/*MPa	*B/*MPa	*n*	*m*	*c*	*T*_m_*/*K
Numerical value	7.85	260	890	0.5	0.94	0.05	1793

The Johnson–Cook material model, well-suited for large-deformation simulations under high strain rates, is employed to characterize the material response under explosive impact. The constitutive equation is as follows:

3 $$\sigma_{eq} = \left( {A + B\varepsilon_{{\mathrm{eq}}}^{n} } \right)\left( {1 + C\ln \dot{\varepsilon }_{{\mathrm{eq}}}^{*} } \right)\left( {1 - T^{*m} } \right)$$

In Eq. (3):${\sigma }_{eq}$ is the equivalent stress; *A* is the initial yield stress at low strain; *B* is the hardening constant;${\varepsilon }_{eq}$ is the equivalent plastic strain; ${\varepsilon }_{eq}^{*}$ is the dimensionless equivalent plastic strain rate; *n* is the hardening index; *C* is the strain rate constant; *m* is the material intrinsic property constant; ${T}^{*}$ is the dimensionless temperature.

**Fig. 2:**
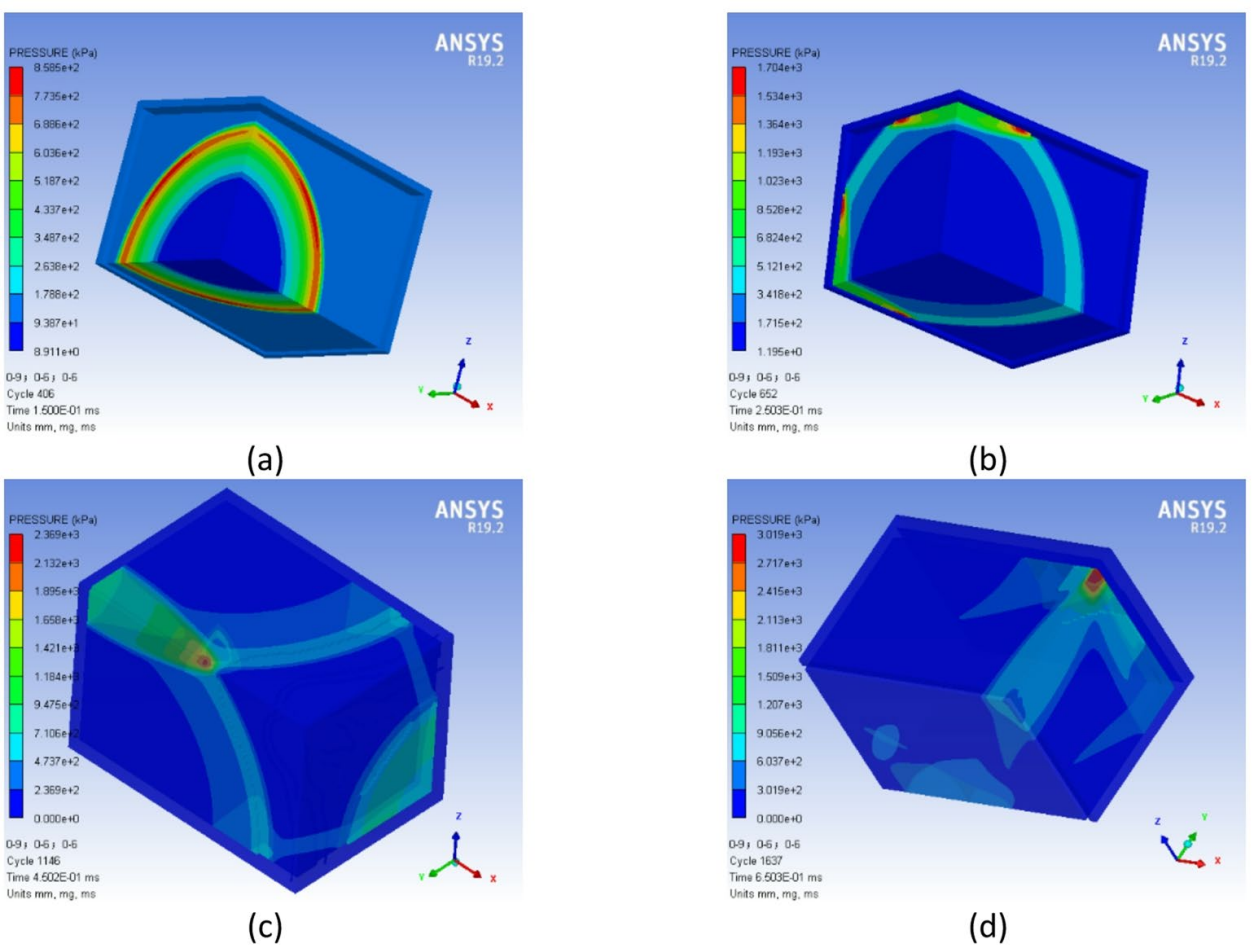
Propagation pressure contours of internal explosion shock waves.

To verify the accuracy of the established numerical model, simulation calculations are performed under the experimental conditions described in literature^23^. The accuracy of the numerical model is validated by comparing experimental measurements with simulation results. Figure 2 shows the propagation process of shock waves generated by the detonation of 25 g explosives inside the cabin, where the red area represents the outermost wavefront surface of the shock wave.

The evolution of shock wave loading in the cabin can be summarized as follows: After explosive detonation, before contacting the cabin inner wall, the explosion shock wave expands in a three-dimensional axisymmetric manner. The outermost red region represents the shock wave front. Due to the equal distance between the charge and the upper and lower walls, the shock wave expansion is symmetric, resulting in identical pressure distributions on both walls. As shown in Fig. 2a, at *t* = 0.15 ms, the explosion shock wave first reaches the wall and undergoes normal reflection. Subsequently, other points on the bulkhead are successively loaded by the shock wave, experiencing regular oblique reflection and Mach reflection. With time, constrained by the surrounding walls, the shock wave undergoes multiple reflections in the confined space. Figure 2b shows that at *t* = 0.25 ms, obvious Mach waves are formed due to irregular reflection from the wall, and the initial shock wave converges with the Mach reflection wave, resulting in a prolonged action time. At *t* = 0.45 ms(Fig. 2c), the explosion shock wave generates localized pressure convergence at the junction of two walls and propagates continuously along the prism direction, leading to a sharp increase in overpressure. At *t* = 0.65 ms(Fig. 2d), the explosion shock wave reaches the cabin corners, forming secondary pressure convergence in the three-wall corner region.

**Fig. 3:**
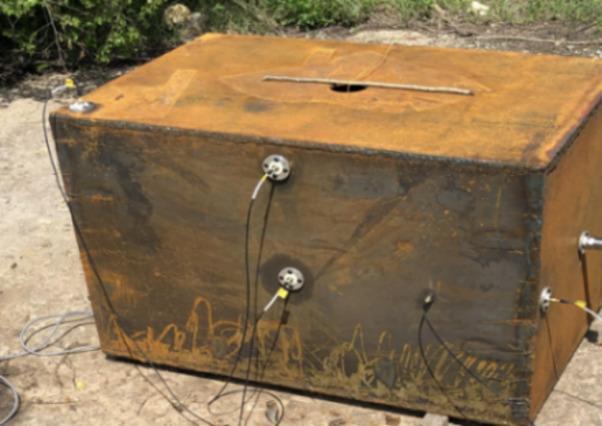
Test site layout.

The test cabin material in literature^23^ is Q235 steel with a wall thickness of 16 mm. Spherical TNT bare charges are used as explosives. The data acquisition system includes a Sichuan Top Measurement and Control Technology Nuxi-1008 high-speed data acquisition instrument connected to high-frequency piezoelectric sensors. The sampling frequency of the shock wave load signal channel is 1 MHz, with an external trigger mode. The pressure sensor model is CY-YD-205 produced by Jiangsu Sunergy Electronic Technology Co., Ltd., with parameters: measuring range 0 ~ 10 MPa, output voltage 0 ~ 5 V, operating temperature − 40 ~ 150 °C, and response frequency 100 kHz. The test site layout is shown in Fig. 3.

**Fig. 4:**
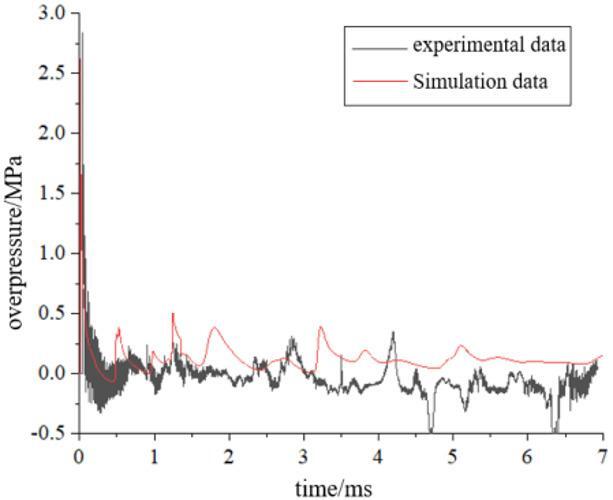
Comparison between numerical simulation and experimental results.

Figure 4 presents the comparison between numerical simulation and test results of bulkhead normal reflection under 25 g explosive loading. The numerical simulation results intuitively reflect the multiple pulse phenomenon of pressure loading. The peak overpressure of the shock wave load in this case is 2.62 MPa, with a deviation of 7.487% between the numerical simulation value and the test measurement. This indicates that the proposed numerical simulation method exhibits good applicability for calculating shock wave loads in cabin explosions.

### Calculation model for shock wave peak overpressure in the cabin

**Fig. 5:**
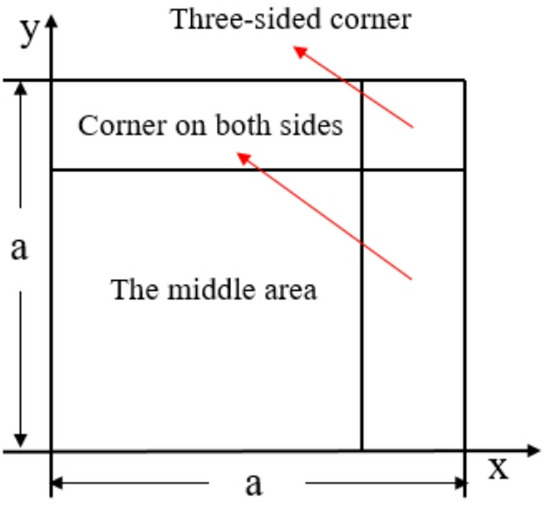
Bulkhead regional division.

In confined space explosions, constrained by the cabin walls, shock waves undergo multiple reflections, superpositions, and convergence. After the shock wave load stabilizes, a long-duration quasi-static pressure load is formed inside the cabin. This study focuses on square bulkheads. To accurately obtain the peak overpressure of reflected shock waves in the cabin, the bulkhead is divided into three types of regions based on the regional boundary conditions specified in literature^24^: Region I (central area far from boundaries), Region II (two-sided corner convergence area), and Region III (three-sided corner area). The regional division of the bulkhead is illustrated in Fig. 5.

The spatial distribution of shock wave loads is studied via numerical simulation, and a calculation model is established. The peak loads of observation points on the cabin wall in different reflection regions are fitted. The numerical simulation working conditions are listed in Table 3, and the peak overpressures of shock waves in different regions under each working condition are organized to fit the equations for reflected shock wave overpressure in the cabin.

**Table 3:** Numerical simulation working conditions.

Serial number	Cabin size/m	Charge mass/kg				
1	2.5 × 2.5 × 2.5	0.1	0.5	1	2	3
2	5 × 4 × 2.5	0.1	0.5	1	2	3
3	4 × 3 × 2.5	0.1	0.5	1	2	3
4	4 × 2 × 2.5	0.1	0.5	1	2	3
5	5 × 2 × 2.5	0.1	0.5	1	2	3
6	6 × 2 × 2.5	0.1	0.5	1	2	3
7	7 × 2 × 2.5	0.1	0.5	1	2	3
8	8 × 2 × 2.5	0.1	0.5	1	2	3

A calculation model for the peak overpressure of reflected shock waves in the cabin is established, with the basic form defined as:

4 $$\, p_{{\mathrm{r}}} { = }f{(}z{) = }\frac{{A_{{1}} }}{{\mathrm{Z}}} + \frac{{A_{2} }}{{Z^{2} }} + \frac{{A_{3} }}{{Z^{3} }} + \frac{{A_{4} }}{{Z^{4} }}$$

After finalizing the form of the computational model, its parameters are calibrated against the numerical simulation data. This process yields the equations for the peak overpressure of the reflected shock wave in the central wall region, the two-sided corner region, and the three-sided corner region:

Central area:

5 $$P_{{\mathrm{r}}} = \frac{10.43612}{Z} - \frac{44.8483}{{Z^{2} }} + \frac{63.61725}{{Z^{3} }} - \frac{25.21416}{{Z^{4} }}$$

Two-sided corner area:

6 $$P_{{\mathrm{r}}} = \frac{6.01962}{Z} - \frac{29.07216}{{Z^{2} }} + \frac{51.13876}{{Z^{3} }} - \frac{18.54882}{{Z^{4} }}$$

Three-sided corner area:

7 $$P_{{\mathrm{r}}} = \frac{44.2234}{Z} - \frac{220.60583}{{Z^{2} }} + \frac{355.7384}{{Z^{3} }} - \frac{158.24074}{{Z^{4} }}$$

where: *P*_r_ is the peak overpressure; *Z* is the scaled distance, $Z=R/\sqrt[3]{W}$, *R* is the detonation distance, *W* is the explosive mass.

### Simplified calculation model for internal explosive loads

**Fig. 6:**
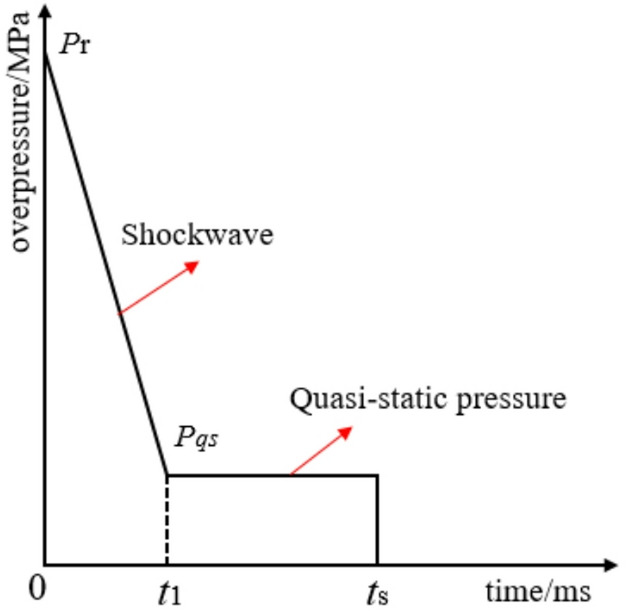
Simplified schematic of internal explosion load.

Due to the complexity of the internal explosion environment, the theoretical calculation process becomes excessively intricate, necessitating the simplification of cabin loads^25^. The key parameters of cabin explosion loads include: shock wave peak load *P*_r_, shock wave action time *t*_1_, quasi-static load peak *P*_qs_, and quasi-static action time *t*_2_. In this study, the cabin explosion load is simplified based on impulse equivalence, as illustrated in Fig. 6.

It should be noted that the shock wave pressure within the cabin is spatially non-uniform, with significant amplification at the corners. However, for the global plastic deformation of ductile metal plates, the response is governed by the total energy input rather than local pressure peaks. Based on the impulse equivalence principle, the spatially varying load can be effectively simplified into an equivalent uniform load.

However, this simplification has limitations. It is primarily applicable to single-compartment structures. For multi-compartment scenarios or structures with complex boundary configurations, where wave diffraction and venting effects significantly alter the load distribution, this simplified model may underestimate local responses and would require further refinement or coupled CFD-FEM simulations. Nevertheless, for the purpose of predicting the maximum central deflection of a single bulkhead, this method demonstrates sufficient engineering accuracy (as verified in "Test result analysis" section).

To simplify the cabin explosion load, the basic parameters of the load must first be determined. Given the rapid decay rate of shock wave loads in the cabin explosion environment, the decay curve is simplified to a linear form:

8 $$P_{{\left( {t_{1} } \right)}} = \frac{{P_{qs} - P_{\mathrm{r}} }}{{t_{1} }}t + P_{\mathrm{r}}$$

In Eq. (8): *P*_r_ is the peak overpressure of reflected shock wave in the cabin (MPa); *P*_qs_ is the peak overpressure of quasi-static pressure (MPa).

The effective shock wave action time $t_{1}$ is:

9 $$t_{1} = B \cdot \sqrt R \cdot \sqrt[6]{W}$$

In Eq. (9): *B* = 1.3 ~ 1.5; *R* is the detonation distance (m); *W* is the mass of TNT (kg).

The quasi-static pressure overpressure is calculated using the Carlson formula:

10 $$P_{qs} = 1.3W/V$$

In Eq. (10): *W* is the TNT mass (kg); *V* is the confined space volume (m^3^).

Based on the structural saturation response characteristics^26^, there exists a saturation response time *t*_s_ of the structure under impact loading:

11 $$t_{s} = \lambda L\sqrt {\frac{\rho }{{\sigma_{0} }}}$$

In Eq. (11): ${t}_{s}$ is the saturation response time of the plate structure (ms), which represents the duration required for the structure to reach its maximum plastic deformation under pulse loading^22^; *L* is the side length of the target bulkhead (m); $\rho$ is the material density (g/cm^3^); ${\sigma }_{0}$ is the material yield strength (MPa); $\lambda$ = 16.0 ~ 17.5 (dimensionless coefficient).

The effective quasi-static action time ${t}_{2}$ is expressed as:

12 $$t_{2} = t_{s} - t_{1}$$

Simplifying the cabin explosion load based on impulse equivalence, the specific impulse expressions of the internal explosion load are derived as:

13 $$I_{1} = \int_{0}^{{t_{1} }} {P_{{\left( {t_{1} } \right)}} } dt$$

14 $$I_{2} = \int_{{t_{1} }}^{{t_{2} }} {P_{qs} } dt$$

where ${I}_{1}$ is the shock wave specific impulse (MPa·ms); and ${I}_{2}$ is the quasi-static specific impulse (MPa·ms).

## Theoretical analysis of bulkhead deflection and deformation

### Solution of bulkhead plastic deformation energy

**Fig. 7:**
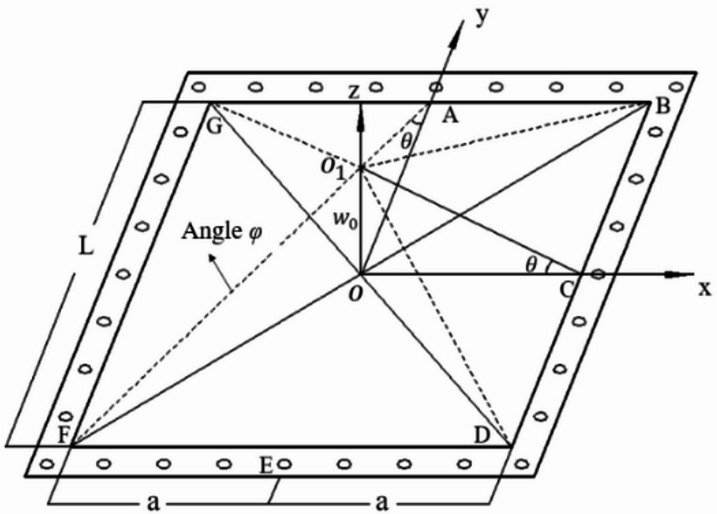
Schematic of bulkhead plastic deformation.

The bulkhead is simplified as a square metal plate with length and width *L* and thickness *H.* Under cabin explosion conditions, the bulkhead is subjected to shock waves and quasi-static loads, resulting in bending deformation. Assuming the bulkhead behaves as an ideal rigid-plastic material with negligible elastic deformation, the internal explosion load acts on the bulkhead in the form of energy, endowing the bulkhead with initial kinetic energy. During the deformation process, the kinetic energy is gradually converted into the plastic deformation energy of the bulkhead, ultimately reaching equilibrium. For the convenience of theoretical calculation, a coordinate system is established with the geometric center of the bulkhead as the origin. The plastic hinge lines formed on the bulkhead under internal explosion loading are illustrated in Fig. 7.

Assuming the explosion-generated internal load is uniformly distributed on the bulkhead, the bulkhead undergoes large plastic deformation, forming plastic hinge lines at the boundary and inside the bulkhead. The deflection surface of the bulkhead under load is approximated as a sinusoidal surface, described by the deflection function^27^:

15 $$\left\{ {\begin{array}{*{20}c} {u = u_{0} \sin \frac{\pi x}{a}\cos \frac{\pi y}{{2a}}} \\ {v = v_{0} \sin \frac{\pi y}{a}\cos \frac{\pi x}{{2a}}} \\ {w = w_{0} \cos \frac{\pi x}{{2a}}\cos \frac{\pi y}{{2a}}} \\ \end{array} } \right.$$

In Eq. (15): *u, v, w* are the displacements of any point on the bulkhead in the *x*, *y*, and *z* directions; ${u}_{0}$ and ${\nu }_{0}$ are the maximum displacement of the bulkhead in the *x* and *y* directions;${w}_{0}$ is the maximum displacement of the bulkhead center in the *z* direction; *a* is half the side length of the bulkhead.

In the plane stress state, the strain components of the bulkhead in the *x* and *y* directions are respectively:

16 $$\left\{ {\begin{array}{*{20}c} {\varepsilon_{x} = \frac{\partial u}{{\partial x}} + \frac{1}{2}\left( {\frac{\partial w}{{\partial x}}} \right)^{2} } \\ {\varepsilon_{y} = \frac{\partial v}{{\partial y}} + \frac{1}{2}\left( {\frac{\partial w}{{\partial y}}} \right)^{2} } \\ {\gamma_{xy} = \frac{\partial u}{{\partial y}} + \frac{\partial v}{{\partial x}} + \left( {\frac{\partial w}{{\partial x}}} \right)\left( {\frac{\partial w}{{\partial y}}} \right)} \\ \end{array} } \right.$$

The total plastic deformation energy of the compartment wall *E* is mainly composed of the plastic hinge line bending deformation energy *E*_1_ at the boundary of the compartment wall, the plastic hinge line bending deformation energy *E*_2_ within the compartment wall and the elongation deformation energy ${E}_{3}$ within the compartment wall surface, then there are:

17 $$E = E_{1} + E_{2} + E_{3}$$

When the bulkhead undergoes large deformation, the bending deformation energy of boundary plastic hinge lines *E*_1_ is:

18 $$E_{1} = \frac{{16\int_{0}^{{\mathrm{a}}} {M_{0} \theta dx} }}{\sqrt 3 }$$

In Eq. (18):${M}_{0}$ is the plastic ultimate bending moment of the bulkhead, ${M}_{0}={\sigma }_{0}{H}^{2}/4$; ${\sigma }_{0}$ is the yield strength of the bulkhead material, considering the strain rate effect, the dynamic yield strength ${\sigma }_{d}$ is used instead of the static yield strength ${\sigma }_{0}$ . $\theta$ is the rotation angle of the plastic hinge line at the bulkhead boundary, $\theta ={\omega }_{0}\frac{\pi }{2a}\mathit{cos}\frac{\pi x}{2a}$.

Rearranging Eq. (18) gives:

19 $$E_{1} = \frac{{16\int_{0}^{{\mathrm{a}}} {M_{0} \theta dx} }}{\sqrt 3 } = \frac{{4\sqrt 3 \sigma_{0} H^{2} w_{0} }}{3}$$

The bending deformation of the bulkhead leads to the formation of energy-dissipating plastic hinge lines on its surface. Therefore, this energy component must be included in the calculation. The relative angle of rotation at a plastic hinge line $\varphi$ is given by:

20 $$\varphi = \frac{{\sqrt 2 w_{0} }}{a}$$

Based on symmetry, the bending deformation energy of internal plastic hinge lines is:

21 $$E_{2} = \frac{2}{\sqrt 3 }M_{0} \left( {4\sqrt 2 \cdot \varphi \cdot {\mathrm{a}}} \right) = \frac{{4\sqrt 3 \sigma_{0} H^{2} w_{0} }}{3}$$

The total bending deformation energy of plastic hinge lines is:

22 $$E_{1} + E_{2} = \frac{{8\sqrt 3 \sigma_{0} H^{2} w_{0} }}{3}$$

When the plastic deformation of the bulkhead under internal explosion loading is tens of times the wall thickness, the membrane force effect dominates over the bending moment effect. According to the Mises yield criterion, the in-plane stretching deformation energy is:

23 $$E_{3} = H\int_{ - a}^{a} {\int_{ - a}^{a} {\left( {\sigma_{0} \varepsilon_{x} + \sigma_{0} \varepsilon_{y} + \frac{{\sigma_{0} }}{\sqrt 3 }\gamma_{xy} } \right)dxdy} }$$

Substituting Eqs. (15) and (16) into Eq. (23) yields:

24 $$E_{3} = \frac{{H\pi^{2} \sigma_{0} w_{0}^{2} }}{4}$$

The total plastic deformation energy of the bulkhead under internal explosion loading is:

25 $$E = \frac{{8\sqrt 3 \sigma_{0} H^{2} w_{0} }}{3} + \frac{{H\pi^{2} \sigma_{0} w_{0}^{2} }}{4}$$

### Solution of bulkhead initial kinetic energy

The kinetic energy of the bulkhead is expressed as:

26 $$T = \frac{1}{2}mv^{2}$$

In Eq. (26): *m* is the mass of the bulkhead in the plastic deformation region,$m=4{a}^{2}\rho H$; *v* is the initial velocity of the bulkhead in the plastic deformation region.

The initial velocity *v* of the bulkhead is derived using the momentum theorem:

27 $$v = \frac{{I_{1} + I_{2} }}{\rho H}$$

In Eq. (27):${I}_{1}$, ${I}_{2}$ are the ratio impulse of shock wave and quasi-static load (MPa·ms); $\rho$ is the bulkhead material density (kg/m^3^); *H* is the bulkhead thickness (mm).

Then the initial kinetic energy *T* of the bulkhead can be expressed as:

28 $$T = \frac{{2a^{2} \left( {I_{1} + I_{2} } \right)^{2} }}{\rho H}$$

### Calculation of bulkhead center deflection

Based on the law of energy conservation:

29 $$\frac{{2a^{2} \left( {I_{1} + I_{2} } \right)^{2} }}{\rho H} = \frac{{8\sqrt 3 \sigma_{0} H^{2} w_{0} }}{3} + \frac{{H\pi^{2} \sigma_{0} w_{0}^{2} }}{4}$$

Rearranging Eq. (29) gives the bulkhead deflection solution equation:

30 $$Aw_{0}^{2} + Bw_{0} + C = 0$$

In Eq. (30):$$A=\frac{H{\pi }^{2}{\sigma }_{0}}{4}$$,$$B=\frac{8\sqrt{3}{\sigma }_{0}{H}^{2}}{3}$$,$$C=-\frac{2{a}^{2}({I}_{1}+{I}_{2}{)}^{2}}{\rho H}$$.

The deflection at the bulkhead center is:

31 $$w_{0} = \frac{{ - B \pm \sqrt {B^{2} - 4AC} }}{2A}$$

Given the TNT mass, detonation distance, bulkhead material parameters, and geometric dimensions, the bulkhead center deflection can be calculated using Eq. (31).

## Internal explosion test validation

### Test program design

**Fig. 8:**
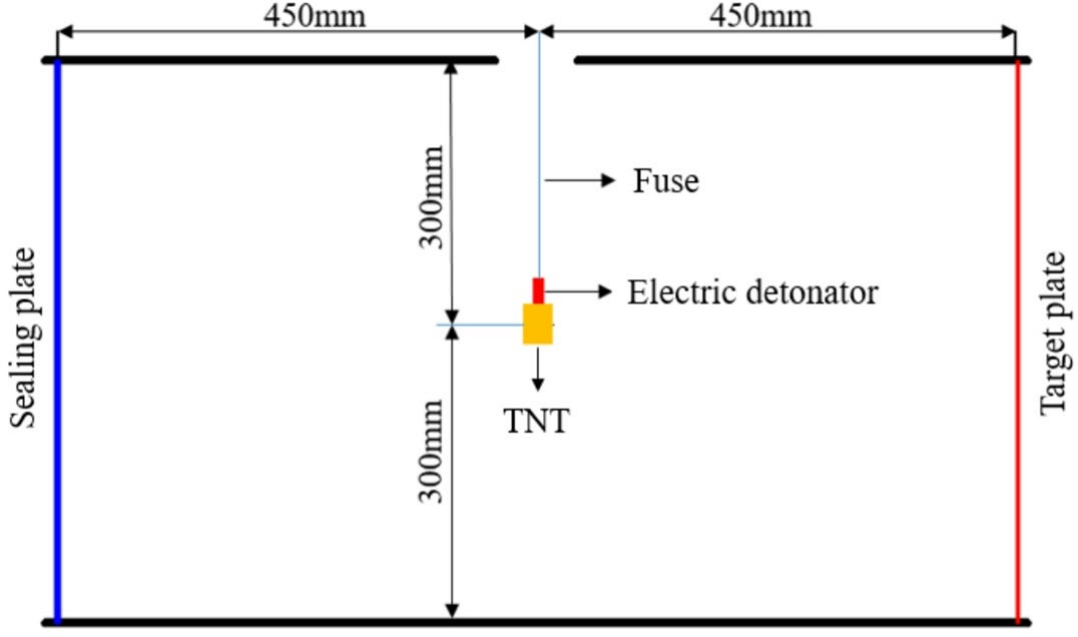
Test layout diagram.

A cabin structure with openings at both ends is adopted in the test. A confined space is formed by fixing target plates at both ends of the cabin to simulate the internal explosion environment. The test aims to verify the accuracy of the proposed bulkhead deflection deformation calculation method under internal explosion loading, with the specific layout illustrated in Fig. 8.

**Fig. 9:**
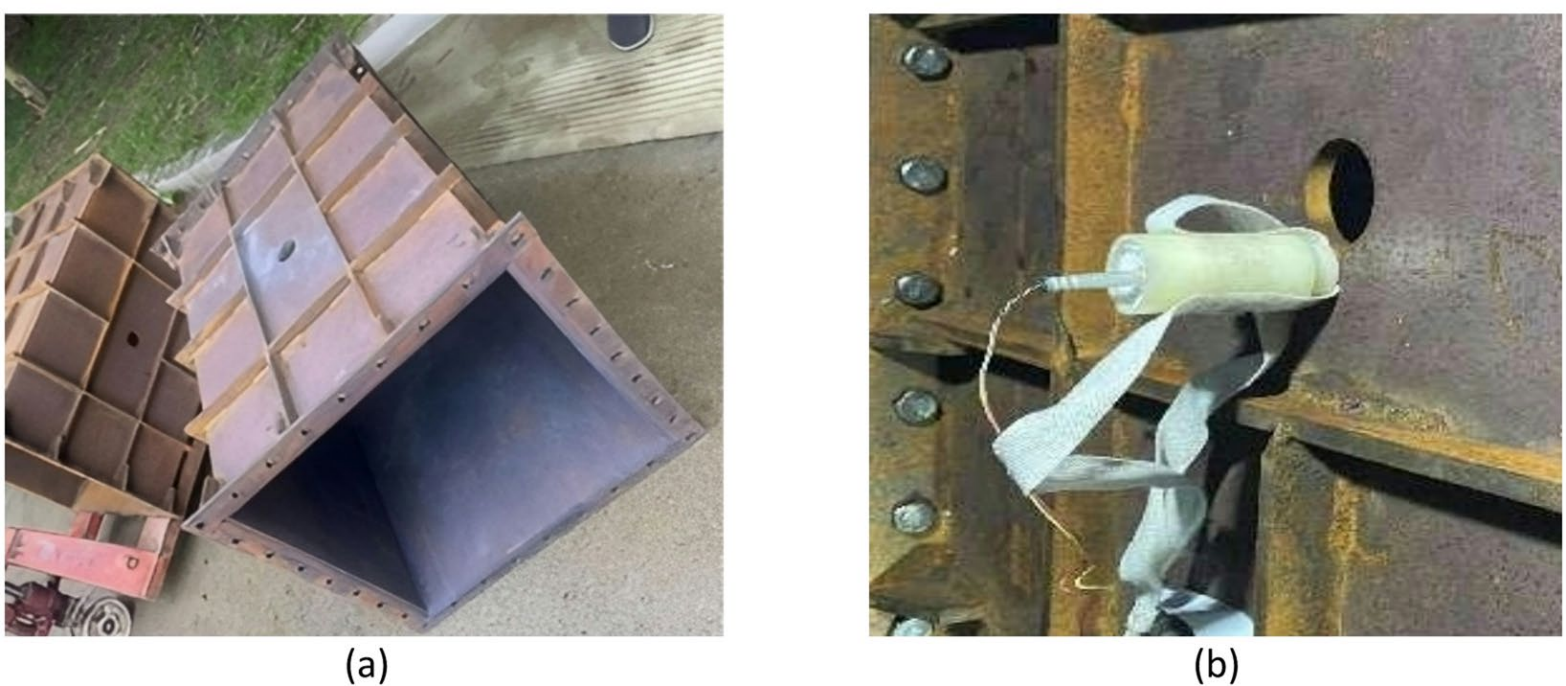
Schematic of the test cabin structure.

The internal explosion cabin structure used in the test is shown in Fig. 9, with internal dimensions of 900 mm × 600 mm × 600 mm. The cabin is fabricated by welding high-strength 45# steel, with a wall thickness of 10 mm. To prevent cabin structure deformation during the internal explosion test, reinforcing bars are welded around the cabin for reinforcement. A 50 mm-diameter hole is drilled at the top of the cabin for charge placement, and bare explosives are suspended at the cabin center through this hole.

**Fig. 10:**
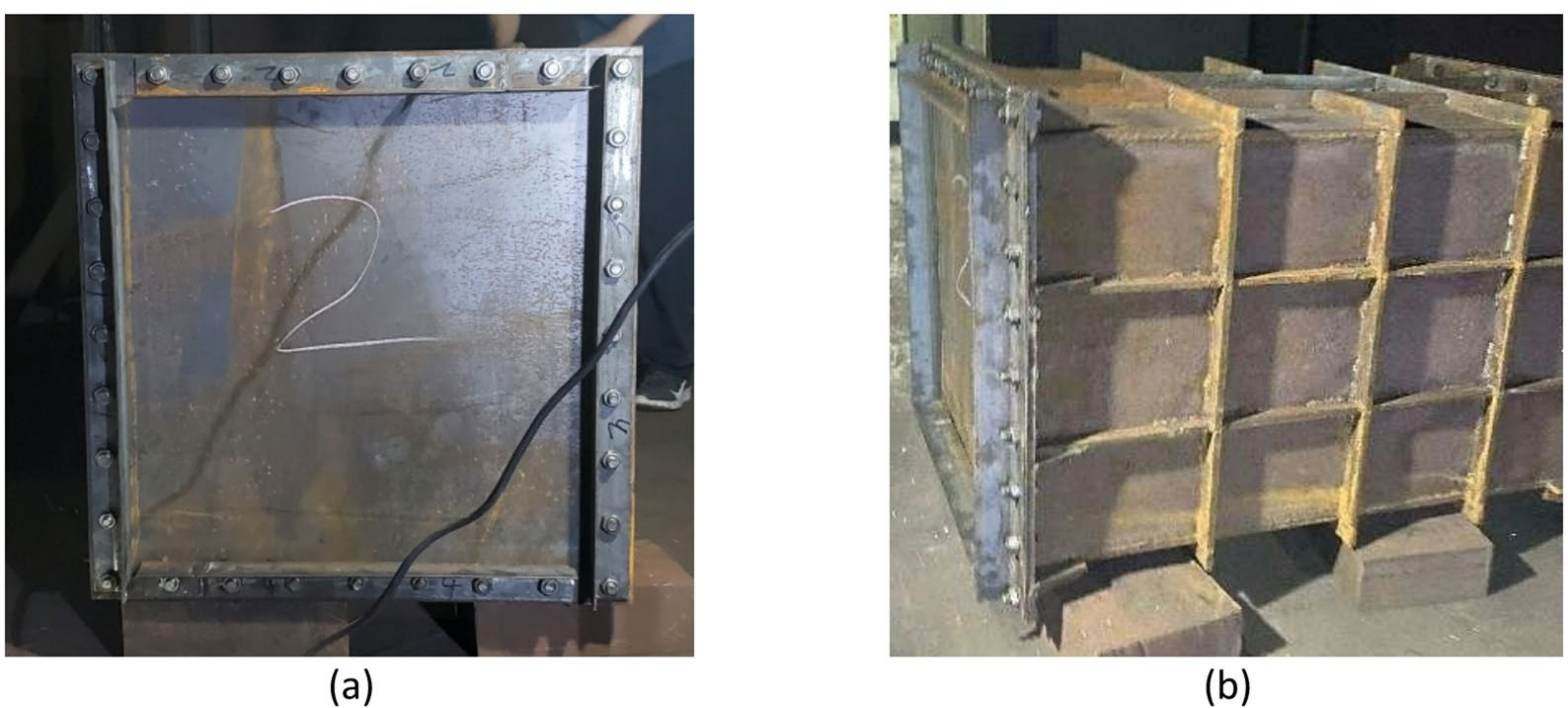
Test site layout.

The target specimens were square Q235 steel plates. To investigate the influence of structural stiffness on the dynamic response, plates with thicknesses of 2 mm and 4 mm were selected for the primary test series. The specimens were secured to the cabin opening using a bolted flange system equipped with 32 bolt holes (18 mm diameter). This fixture method, shown in Fig. 10, was designed to prevent in-plane slippage and approximate a fully clamped boundary condition. The effective loaded area of the target plate was 600 mm × 600 mm. Spherical bare TNT charges were suspended at the geometric center of the cabin to generate the internal blast loading.

### Test result analysis

**Fig. 11:**
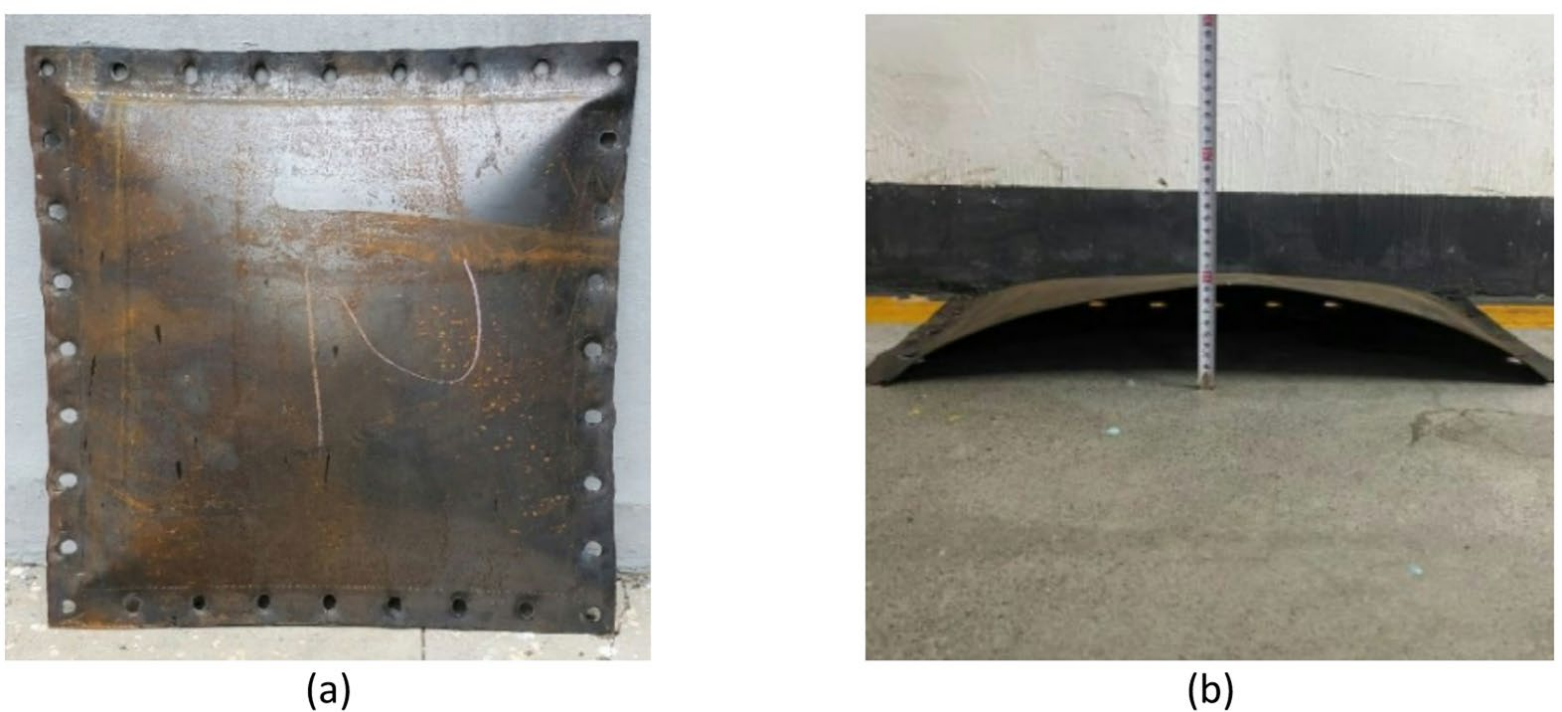
Test results of the target plate.

The test results indicate that the target plate undergoes significant plastic deformation along the load propagation direction, characterized by a large permanent deflection. Figure 11 presents the typical deformation mode of the 2 mm target plate under 80 g TNT explosive loading.

To systematically validate the accuracy and generalizability of the proposed theoretical method, the comparison was extended to include multiple working conditions. In addition to the base experimental case (80 g TNT, 2 mm plate), we incorporated: (1) our own experimental data for a thicker bulkhead (4 mm) under the same 80 g TNT loading; and (2) independent experimental datasets from Li^28^ and Wang^29^. These external cases involved a smaller cabin dimension (500 mm × 500 mm ) and a significantly higher charge mass (135 g TNT).

The theoretical maximum deflections were calculated using the derived Eq. (31) and compared with the experimental measurements. The detailed comparison is summarized in Table 4.

**Table 4:** Comparison between experimental and theoretical calculations results.

Data source	Plate Size /mm	Plate thickness/mm	Explosive mass/g	Test value/mm	Theoretical value/mm	Relative error/%
Present study	600 × 600	2	80	97	110.4	13.8
Present study	600 × 600	4	80	65	73.3	12.7
Li^28^	500 × 500	2	135	80.3	91.5	13.9
Wang^29^	500 × 500	1.8	135	88.6	99.2	11.9

As indicated in Table 4, the relative errors for all validated cases are consistently within 14%, demonstrating the reliable predictive performance of the proposed model. It is observed that the theoretical model exhibits consistent prediction trends across different plate thicknesses (1.8 mm, 2 mm, 4 mm), with no significant deviations from the experimental results. This consistency is attributed to the comprehensive integration of strain-rate effects and quasi-static pressure contributions in the theoretical formulation, which effectively captures the key mechanisms of bulkhead deformation under internal explosion loading. Nevertheless, the consistent agreement across different cabin geometries, plate thicknesses (1.8 mm, 2 mm, 4 mm), and charge masses (80 g, 135 g) confirms that the proposed energy-based method is robust and applicable for engineering rapid assessment of bulkhead deflection under internal explosion loading.

## Conclusions

This study conducted a comprehensive analysis of the plastic dynamic response of ship bulkheads under internal blast loading. Based on a simplified load model and the principle of energy conservation, a theoretical calculation method for predicting the plastic deformation of bulkheads was proposed. The main conclusions are as follows:

- A numerical model for shock wave propagation in cabin explosions was established. The accuracy of the numerical model was validated against experimental data, achieving a relative error of 7.48%, which satisfies engineering accuracy requirements. Based on the numerical simulation results, empirical formulas for calculating the peak shock wave overpressure in different regions of the bulkhead were derived.
- Considering the complexity of confined blast environments, a load simplification method was proposed. The shock wave load and quasi-static pressure within the cabin were simplified based on impulse equivalence, which facilitates theoretical derivation. The effectiveness of this simplification strategy was verified through comparative analysis.
- Based on the structural response characteristics, a theoretical derivation for bulkhead plastic deformation was developed using the energy conservation principle, assuming that the initial kinetic energy acquired from the blast is fully converted into plastic deformation energy. Under the experimental conditions (80 g TNT), the relative error between the theoretical prediction and the experimental measurement was 13.8%. Given the complexity of real-world engineering scenarios, this error margin is acceptable. Consequently, the proposed theoretical formulas provide a reliable and efficient basis for the rapid safety assessment and blast-resistant design of naval ship structures.

## Data Availability

The data that support the findings of this study are available from the corresponding author upon reasonable request.

## List of symbols

*ρ*: Material density of bulkhead
*σ**0*: Yield strength of the material
*σ**d*: Dynamic yield strength
*ρ**0*: Air density
*D*: The dimensionless number, detonation speed
*E*: The internal energy per unit mass
*V*: The relative specific volume of the explosive red product
*W*: Mass of TNT explosive
*λ*: Dimensionless coefficient of saturation time
*B*: Coefficient of shock wave action time
*γ*: Adiabatic index of air
*L*: Side length of the square bulkhead
*a*: Half side length of the bulkhead (a = L/2)
*H*: Thickness of the bulkhead
*σ**0*: Yield strength of the material
*Z*: Scaled distance
*V**c*: Volume of the confined cabin
*Ve*: Relative specific volume of explosive product
*P**r*: Peak overpressure of reflected shock wave
*P**qs*: Peak overpressure of quasi-static pressure
*t**1*: Effective action time of shock wave
*t**s*: Saturation response time of the structure
*t**2*: Effective action time of quasi-static pressure
*I**1*: Specific impulse of shock wave
*I**2*: Specific impulse of quasi-static pressure
*A*, *B*, *R**1*, *R**2*, *w*: Constants
*ω*: Transverse deflection of the bulkhead
*ω**0*: Maximum deflection at the bulkhead center
*u,v*: In-plane displacements in x and y directions
*θ*: Rotation angle of the plastic hinge line
*φ*: Relative rotation angle of internal plastic hinge
*M**0*: Plastic ultimate bending moment per unit length
*E*: Total plastic deformation energy
*E**1*: Bending deformation energy at boundary hinge lines
*E**2*: Bending deformation energy at internal hinge lines
*E**3*: In-plane membrane stretching energy
*T*: Initial kinetic energy of the bulkhead
*v**0*: Initial velocity of the bulkhead
